# Optimizing Optogenetic Activation of Purkinje Cell Axons to Investigate the Purkinje Cell – DCN Synapse

**DOI:** 10.3389/fnsyn.2019.00031

**Published:** 2019-11-22

**Authors:** Kim M. Gruver, Alanna J. Watt

**Affiliations:** ^1^Department of Biology, McGill University, Montreal, QC, Canada; ^2^Integrated Program in Neuroscience, McGill University, Montreal, QC, Canada

**Keywords:** optogenetics, electrophysiology, Purkinje cells, cerebellum, action potentials, deep cerebellar nuclei, axon

## Abstract

Optogenetics is a state-of-the-art tool for interrogating neural circuits. In the cerebellum, Purkinje cells serve as the sole output of the cerebellar cortex where they synapse on neurons in the deep cerebellar nuclei (DCN). To investigate the properties of this synaptic connection, we sought to elicit time-locked single action potentials from Purkinje cell axons. Using optical stimulation of channelrhodopsin-2 (ChR2)-expressing Purkinje cells combined with patch-clamp recordings of Purkinje cells and DCN neurons in acute cerebellar slices, we determine the photostimulation parameters required to elicit single time-locked action potentials from Purkinje cell axons. We show that axons require longer light pulses than somata do to elicit single action potentials and that Purkinje cell axons are also more susceptible to light perturbations. We then demonstrate that these empirically determined photostimulation parameters elicit time-locked synaptic currents from postsynaptic cells in the DCN. Our results highlight the importance of optimizing optogenetic stimulation conditions to interrogate synaptic connections.

## Introduction

Optogenetics is a powerful tool that has transformed the investigation of neural circuits. The ability to genetically target and optically activate distinct cell populations of presynaptic neurons allows for functional circuit mapping which has refined our understanding of the brain (Huber et al., 2008; Cruikshank et al., 2010; Pfeffer et al., 2013). Genetically targeted opsins distribute throughout cell membranes and can be detected in all cellular compartments, including the soma, dendrites, and axons (Nagel et al., 2003; Boyden et al., 2005; Lewis et al., 2009). Light pulses can thus be focused onto subcellular compartments to elicit neuronal activity that originates locally (Petreanu et al., 2007; Jackman et al., 2014). For example, targeting axons with focal optical stimulation can be an effective means by which to probe connectivity, especially in acute slices where presynaptic axons are preserved even if their soma is lesioned. However, this approach raises the question of whether focal stimulation of a neuron’s axon requires different conditions than focal stimulation of its soma. This is important to address given that there are several recent reports showing that focal axonal stimulation with inhibitory optogenetic tools paradoxically produces excitation rather than inhibition (Mahn et al., 2016; Messier et al., 2018). These studies highlight the importance of empirically testing conditions for optogenetic experiments.

Cerebellar Purkinje cells carry information from the cerebellar cortex via synapses made onto neurons in the deep cerebellar nuclei (DCN) (Palay and Chan-Palay, 1974; Person and Raman, 2012). Previous studies have demonstrated that this connection can be investigated with Channelrhodopsin-2 (ChR2), since the synaptic responses elicited optogenetically resemble those elicited from extracellular electrical stimulation (Jackman et al., 2014). However, the parameters to elicit action potentials optogenetically can differ with different equipment, for example with a LED versus a laser. To study the synaptic properties of the Purkinje cell – DCN neuron connection optogenetically, we first need to understand how to elicit well-timed single action potentials reliably from Purkinje cell axonal stimulation. Here, we determine the experimental conditions necessary to reliably activate Purkinje cells using a patterned illuminator with a LED light source. We find that focal illumination of Purkinje cell axons requires longer light pulses than somata, and that axons are more susceptible to perturbations from ambient light. Finally, we show that these empirically determined conditions enable us to elicit well-timed synaptic responses in DCN neurons.

## Materials and Methods

### Animals

Transgenic mice hemizygous for Purkinje cell-specific Cre [strain B6.Cg-Tg(Pcp2-cre)3555Jdhu/J; stock number: 010536; *PCP2-Cre*] and mice homozygous for Channel- rhodopsin-2/H134R fused with enhanced YFP [strain: B6;129S-Gt(ROSA)26Sor^tm32(CAG–COP4*H134R/EYFP)Hze^/J; stock number 012569; *Ai32*], or ChR2(H134R)-EYFP, were acquired from The Jackson Laboratory (Bar Harbor, ME, United States) and bred to produce hemizygous *PCP2-Cre/Ai32* mice expressing modified ChR2 in Purkinje cells (Jackman et al., 2014). All animal procedures were approved by the McGill Animal Care Committee, in accordance with guidelines established by the Canadian Council on Animal Care.

### Acute Slice Preparation

Slices were prepared as described previously (Jayabal et al., 2017; Ady et al., 2018). Male and female mice (P20 to P31) were deeply anesthetized with isoflurane and decapitated. Brains were removed and immediately placed in ice-cold artificial cerebrospinal fluid (ACSF; in mM: 125 NaCl, 2.5 KCl, 2 CaCl_2_, 1 MgCl_2_, 1.25 NaH_2_PO_4_, 25 NaHCO_3_, and 25 glucose, bubbled with 95% O_2_–5% CO_2_ to maintain pH at 7.3; osmolality ∼317 mOsm) for Purkinje cell experiments, or partial sucrose replacement slicing solution (in mM: 50 NaCl, 2.5 KCl, 0.5 CaCl_2_, 10 MgCl_2_, 1.25 NaH_2_PO_4_, 25 NaHCO_3_, 25 glucose, and 111 sucrose bubbled with 95% O2–5% CO_2_ to maintain pH at 7.3; osmolality ∼317 mOsm) for DCN experiments. Chemicals were purchased from Sigma-Aldrich (Oakville, ON, Canada) and/or Fisher Scientific (for CaCl_2_ and MgCl_2_; Toronto, ON, Canada). Parasagittal slices of cerebellar vermis and paravermis were cut using a Leica VT 1000S vibrating blade microtome at a thickness of 200 μm. All slices were then incubated in ACSF at 37°C for 30–45 min, and subsequently stored at room temperature for up to 6 h. Slices were typically stored in a chamber that minimized light exposure. However, for ambient light experiments, slices were stored in ACSF in a clear glass chamber in a laboratory with bright overhead lights, and were illuminated with a halogen lamp to visualize Purkinje cells in acute slices. In the “ambient light” condition, slices were exposed to a continuous spectrum of white background light.

### Imaging

Slices were imaged with a custom two-photon microscope equipped with a Ti:Sapphire laser (MaiTai; Spectra Physics, Santa Clara, CA, United States) tuned to 890 nm and image stacks (1 μm z-step) were acquired with ScanImage running in MATLAB (Mathworks, Natick, MA, United States) (Pologruto et al., 2003). Maximal intensity projections of image stacks were generated in ImageJ (US National Institutes of Health^1^).

### Electrophysiology

Borosilicate patch pipettes (2–9 MΩ) were pulled with a P-1000 puller (Sutter Instruments, Novato, CA, United States). For current-clamp experiments in Purkinje cells, the internal solution contained (in mM): 130 potassium gluconate, 0.5 EGTA, 10 HEPES, 4 Mg-ATP, 0.4 Na-GTP, 10 NaCl, 10 KCl, with 286 mOsm and pH 7.3 (adjusted with KOH). For voltage-clamp experiments in DCN neurons, the internal solution contained (in mM): 150 potassium gluconate, 3 KCl, 10 HEPES, 0.5 EGTA, 3 Mg-ATP, 0.5 GTP tris salt, 5 phosphocreatine-(di)tris, with 297 mOsm and pH 7.2 (adjusted with KOH). Recordings were acquired with a Multiclamp 700B amplifier (Molecular Devices, Sunnyvale, CA, United States) on a SliceScope Pro 3000 microscope (Scientifica, Uckfield, United Kingdom) from neurons in slices maintained at a temperature of 34°C ± 1°C and bathed with oxygenated ACSF. Purkinje cells whose resting membrane potential was > −40 mV were excluded from analysis. For voltage-clamp recordings in DCN neurons, cells were voltage-clamped to −60 mV, and R_in_ and resting membrane potential were monitored. Recordings in which the R_in_ changed more than 25% were excluded from analysis. Data acquisition and analyses were performed using custom IGOR Pro acquisition and data analysis software (Sjöström et al., 2001) (Wavemetrics, Portland, OR, United States).

### Optical Stimulation

Slices expressing ChR2 were optically stimulated using a Polygon400E patterned spatial illuminator with a 470 nm LED light source (Mightex, Toronto, ON, Canada), through a 60X water-immersion objective (Olympus LUMPLFLN60XW, Tokyo, Japan). Visually identified regions of interest for photostimulation were delineated using PolyScan2 software (Mightex). Photostimulation was induced while patch-clamping the soma of either Purkinje cells or DCN neurons. We used a 40 × 40 μm blue square light pulse with an estimated focal plane power density of 100 mW/mm^2^ for both axonal and somatic photostimulation, or in some cases, circular light pulses (∼20 μm diameter) were used for somatic stimulation. For axonal photostimulation during Purkinje cell experiments, the area illuminated was 120–200 μm from the Purkinje cell soma. This distance varied due to variation in the thickness of the granule cell layer, but was always in the white matter close to the recorded Purkinje cell. For axonal photostimulation during DCN experiments, the area illuminated was ∼200 μm from the DCN neuron soma, in the white matter adjacent to the DCN. Interstimulus intervals were 15 s for evoking action potentials from Purkinje cell somata or axons and 20 s for eliciting postsynaptic responses in the DCN.

### Data Analysis

All electrophysiological data were analyzed using custom Igor Pro data analysis software (Watt et al., 2009). Action potential latency was measured as the time in ms from the onset of the light stimulus to the peak of the action potential. For inhibitory postsynaptic currents (IPSCs), the rise time was measured as the time between 20 – 80% of the peak. For Purkinje cell recordings, jitter was measured as the variability (represented as standard deviation, SD) in time from the beginning of the light pulse to the peak of the action potential. For DCN recordings, jitter in the onset of the postsynaptic response was measured as the variability (SD) in time to reach 20% of the peak IPSC.

### Statistics

Mann–Whitney *U* tests were performed using JMP software (SAS, Carey, NC, United States) with the level of significance (α) set at *P* < 0.05. Data are reported as mean ± SEM. For all data, *n* = number of cells and *N* = number of mice.

## Results

**FIGURE 1:**
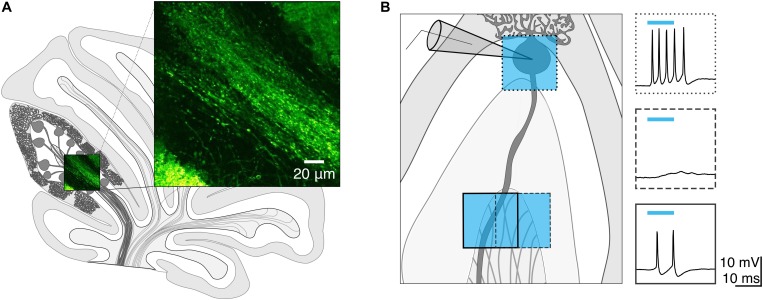
Channelrhodopsin-2 expression in Purkinje cell axons. **(A)** Schematic of sagittal cerebellar slice. Inset is a maximal intensity projection of a two-photon stack showing ChR2(H134R)-EYFP expression (green) in axons in the white matter. **(B)** Left, schematic showing multiple photostimulation regions (blue squares) and somatic recording electrode. Right, representative current-clamp traces show optically evoked action potentials following somatic (box with dotted line outline) and white matter (dashed and solid line outline on boxes) stimulation locations. Shifting the photostimulation location ∼30 μm in the white matter of the cerebellum produced action potentials. Blue bars above traces indicate onset and duration of light pulse.

We wondered whether focal photostimulation of Purkinje cells would result in differential effects depending on the targeted subcellular compartment. To address this, we first confirmed that ChR2 is expressed in Purkinje cell axons of ChR2(H134R)-EYFP mice. Consistent with what has been previously reported (Jackman et al., 2014), we observed robust ChR2 expression in Purkinje cell axons located in the white matter of the cerebellum (Figure 1A).

We next sought to test whether spatially targeted photostimulation of Purkinje cells can be reliably elicited in axons. While this has been demonstrated by others using short light pulses from a laser (Jackman et al., 2014), to our knowledge this has not been characterized from a LED light source. We made whole-cell current-clamp recordings from Purkinje cell somata and injected negative current until we hyperpolarized the cell to silence spontaneous action potentials. Using a spatial illuminator delivering 470 nm light from a LED, we applied a 40 × 40 μm square light pulse either to the soma or to the cerebellar white matter to stimulate axons and recorded antidromic action potentials. To elicit action potentials in the axon, we photostimulated in the white matter while monitoring the somatic recording for the presence of an elicited action potential(s). If no action potential was evoked in one location, we would then parametrically shift our photostimulation location (in 30–40 μm steps) until action potentials were evoked (Figure 1B). If we were unable to elicit action potentials after illuminating multiple stimulation locations, we concluded that the axon of the Purkinje cell was likely cut.

**FIGURE 2:**
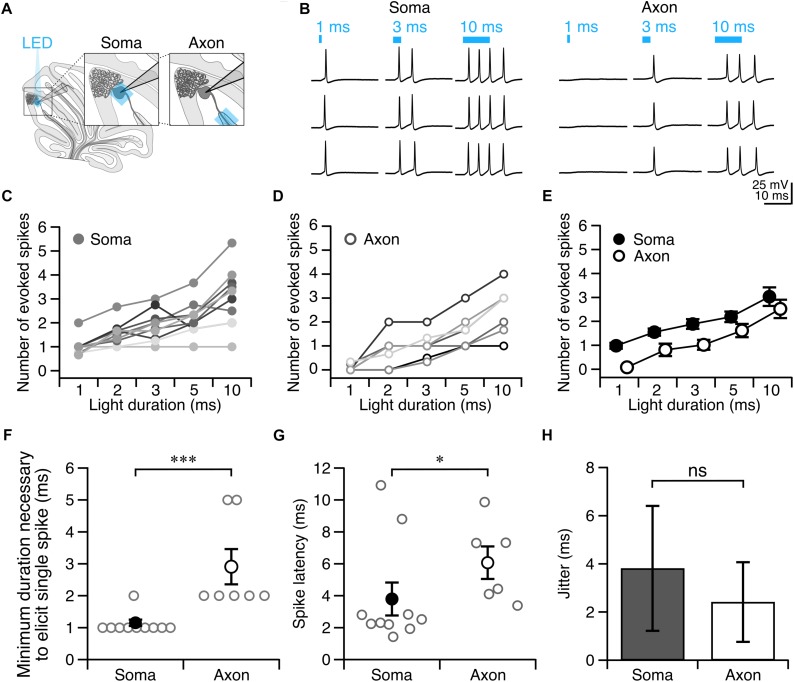
Purkinje cell axons require longer light durations to evoke an action potential than somata. **(A)** Schematic depicting the recording configuration. **(B)** Representative current-clamp traces of optically evoked action potentials evoked following somatic (left) and axonal (right) stimulation. **(C–E)** The number of action potentials evoked for different photostimulus durations. **(C)** Soma, individual cells. **(D)** Axon, individual cells. **(E)** Averages. **(F)** Minimum duration of light stimulus required to elicit a single action potential from each subcellular locus. **(G)** Latency to the first action potential evoked by photostimulus was longer in axons than in soma. **(H)** Jitter of spike latency. Soma: *n* = 10; Axon: *n* = 7. Data represented as mean ± SEM. ns = not significant, *P* > 0.05; ^∗^*P* < 0.05; ^∗∗∗^*P* < 0.001.

Once we had identified a white matter photostimulation location from which we could elicit action potentials (Figure 2A), we tested photostimulus pulses of different durations to explore the conditions required to elicit single action potentials when light was delivered to the soma (Figures 2B(left),C) and axon (Figures 2B(right),D). We found that there was variability in the numbers of action potentials elicited at a given light duration across cells (Figure 2D).

Since our aim was to identify light stimulation conditions that reliably elicit single action potentials across cells, we wanted to avoid eliciting multiple action potentials, although in most cases we were unable to accomplish this without occasional action potential failures (and used a failure cut-off of <33.3%). We found that 1 ms light stimulation reliably elicited single action potentials with somatic illumination (0.98 ± 0.12 spike for 1 ms, 1.55 ± 0.15 spike for 2 ms *n* = 10 cells; *N* = 7 mice; Figure 2E), but not with axonal illumination (0.08 ± 0.06 spike for 1 ms, *n* = 7 cells; *N* = 6 mice; Figure 2E). The optimal light stimulation duration that elicited single action potentials for axonal stimulation was typically 2 or 3 ms for individual cells (2 ms: 0.81 ± 0.26 spike; 3 ms: 1.02 ± 0.21 spike, Figure 2E). We sought to identify the optimal minimal light stimulation to elicit action potentials from the soma and axon for each cell, and found that the average minimal light duration necessary for axons (axon minimal light duration: 2.86 ± 0.55 ms; Figure 2F), was significantly longer than for somata (soma minimal light duration = 1.10 ± 0.10 ms, *n* = 10, *N* = 7; *P* = 0.0003; Figure 2F). This was also longer than what has previously been reported with a laser light source (Jackman et al., 2014). The latency from light onset to the evoked action potential was also significantly shorter for the soma than for the axon (soma: latency = 3.80 ± 1.03 ms; axon: latency = 6.07 ± 1.02 ms; *P* = 0.042; Figure 2G). However, although the latency to fire single action potentials with somatic or axonal photostimulation differed, we found no significant differences in the jitter of evoked spikes (soma: jitter = 3.81 ± 2.59 ms; axon: jitter = 2.42 ± 1.66 ms; *P* = 0.46; Figure 2H), suggesting that photostimulation results in consistently time-locked action potentials from both the axon and soma.

**FIGURE 3:**
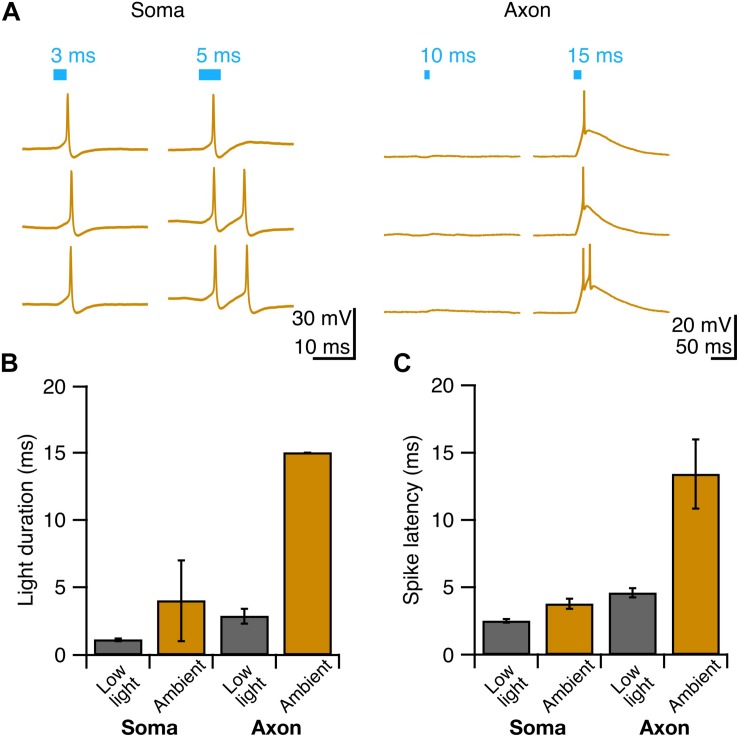
Purkinje cell axons are more vulnerable to suboptimal experimental conditions than cell bodies. **(A)** Representative current-clamp traces of optically evoked action potentials elicited from Purkinje cell somata (left) and axons (right) when exposed to ambient light. **(B)** Axons require longer light durations to elicit single spikes in ambient light than somata. **(C)** The latency to spike after pulse onset for somata and axons exposed to ambient light. Ambient light: Soma: *n* = 3; Axon: *n* = 3.

Since photostimulation of Purkinje cell axons requires longer light pulses to elicit an action potential than somatic stimulation, we wondered whether axons might be more susceptible to light perturbations, such as exposure to background white light that might result in inactivation of ChR2 channels (Lin et al., 2009). To test this, we exposed Purkinje cells to ambient light during slice incubation and recordings, and elicited action potentials as before (Figure 3A, see section “Materials and Methods”). We observed an increase in the pulse duration necessary to evoke a single action potential from axons exposed to ambient light compared to what was observed for experiments performed in low light (*P* = 0.013; Figure 3B). By comparison, we did not find a difference in the pulse duration necessary to reliably elicit a single action potential from the soma between ambient light and low light conditions (*P* = 0.35; Figure 3B). Although the spike latency showed a tendency to increase in the ambient light condition compared to the low light condition for both the soma and axon (Figure 3C), these differences were not significant. These results suggest that Purkinje cell axons are more susceptible to ambient light than somata are, perhaps due to the presumed lower density of ChR2 channels in axons rendering them proportionally more sensitive to photo-inactivation.

**FIGURE 4:**
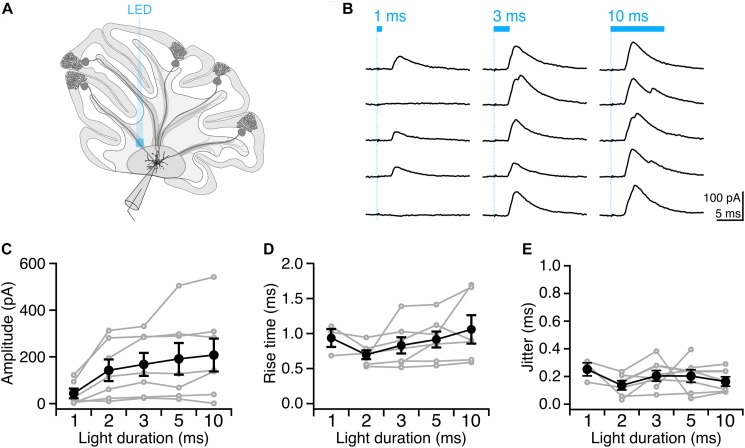
Precisely timed synaptic responses in a DCN neuron with optogenetic Purkinje cell activation. **(A)** Recording configuration. Light pulses were delivered to Purkinje cell axons while performing whole-cell voltage-clamp recordings in DCN neurons. **(B)** Representative traces of IPSCs evoked with durations of light in same location. Blue bar above traces indicates onset and duration of light pulse. **(C)** Average IPSC amplitude across photostimulus durations. **(D)** Average IPSC rise time. **(E)** Jitter of onset of IPSCs. Individual cells, gray. Average, black. *n* = 7.

Having identified conditions that reliably elicit single well-timed action potentials in Purkinje cell axons, we then sought to determine whether this paradigm would allow us to robustly elicit well-timed postsynaptic responses in DCN neurons. After making whole-cell voltage-clamp recordings from DCN neurons (Figure 4A), we stimulated Purkinje cell axons with variable light durations in the white matter ∼200 μm from the patched cell, and recorded evoked IPSCs (Figure 4B). IPSC amplitude increased modestly with increasing photostimulus duration (Figure 4C), which may be due to additional action potentials elicited with longer light stimulation durations (Figures 2D,E), or from additional presynaptic axons being recruited by longer pulses. Rise times of evoked IPSCs were rapid (0.88 ± 0.06 ms, *n* = 7 cells; *N* = 3 mice, Figure 4D), with averages varying <0.2 ms across different stimulation durations, consistent with fast kinetics previously reported for this synapse (Pedroarena and Schwarz, 2003; Pugh and Raman, 2005; Uusisaari and Knöpfel, 2008). The jitter of the onset of postsynaptic response was low for all photostimulus durations, consistent with well-timed action potentials (Table 1 and Figure 4E).

**TABLE 1:** Synaptic properties of evoked IPSCs from DCN neurons.

**Duration**	**1 ms**	**2 ms**	**3 ms**	**5 ms**	**10 ms**
Amplitude (pA)	43.59 ± 20.92	142.80 ± 46.33	168.30 ± 49.18	191.80 ± 67.71	207.90 ± 70.32
Rise time (ms)	0.94 ± 0.13	0.70 ± 0.06	0.83 ± 0.12	0.91 ± 0.11	1.06 ± 0.2
Jitter of onset (ms)	0.25 ± 0.05	0.14 ± 0.03	0.21 ± 0.04	0.20 ± 0.05	0.16 ± 0.03

*Amplitude, rise time, and jitter of the onset of the postsynaptic response (time to 20% of the peak) were determined from the average IPSCs evoked for each photostimulus duration. Amplitude analysis includes failures. *n* = 7.*

We found that with increasing light duration we saw more instances of multi-peak IPSCs (Figure 4B), which is in line with our observation that longer light durations elicit multiple presynaptic action potentials (Figure 2B), but may also reflect the recruitment of additional axons with longer pulses. Based on our empirical results above, we conclude that a 2 or 3 ms photostimulation duration is best suited to reliably elicit well-timed single presynaptic action potentials in Purkinje cell axons in order to investigate the Purkinje cell – DCN synapse.

## Discussion

We determined the light pulse duration from a 470 nm LED required to elicit well-timed single action potentials in Purkinje cell axons in acute sagittal slices from transgenic mice expressing ChR2 in Purkinje cells. We show that axons require longer pulse durations than somata to elicit the same number of action potentials, and that axonal photostimulation causes longer latencies to spike than somatic photostimulation. We also found that axons are more susceptible to perturbation from background light exposure. Finally, we demonstrate that the conditions we have used elicit well-timed single action potentials from Purkinje cell axonal stimulation allow us to elicit robust time-locked synaptic currents in postsynaptic neurons in the DCN. Since several recent studies using inhibitory optogenetic tools have shown that focal photostimulation of somata and axons yields different results, where stimulation of axons can result in paradoxical effects on activity (Mahn et al., 2016; Messier et al., 2018), we set out to confirm whether the conditions required for axonal photostimulation were similar to those for Purkinje cell somatic stimulation from mice transgenically expressing EYFP-fused ChR2(H134R). We found that we could elicit well-timed action potentials in both the soma and axon with focal photostimulation, although axons required longer light pulses, and displayed longer latencies. These light pulses were longer than what has been previously reported using a laser for photostimulation (Jackman et al., 2014).

Since we measured action potentials with a somatic patch pipette recording, we expected action potential latencies to be shorter when evoked from the soma than from the axon. Purkinje cell axons have been estimated to have a conduction velocity of ∼1–10 m/s (Khaliq and Raman, 2005), so given the distances between axonal stimulation location and somatic patch pipette (<200 μm separation), only a small fraction of the increased latency (up to 0.2 ms) should be attributed to the conduction latency arising from the distal site of axonal action potential initiation. Several other factors likely contribute to the increased latency of action potentials arising from axonal stimulation. Purkinje cell axons are myelinated (Ljungberg et al., 2016) and action potentials travel between nodes of Ranvier in the axon. However, given that internodal spacing ranges between 60 and 260 μm (Clark et al., 2005), the area of focal photostimulation is likely to only occasionally overlap with a node of Ranvier. In support of this, internodal spacing of nodes of Ranvier have been shown to be a limiting factor in the induction of action potentials in myelinated axons (Arlow et al., 2013). Since light scattering is increased in lipid-rich tissues such as myelin which is abundant in the cerebellar white matter, lower light intensities likely reach Purkinje cell axons compared to the soma (Mattis et al., 2012). However, although the latency to action potential is longer for axons, the jitter between trials is not significantly different, suggesting that action potentials can be elicited reliably and with high temporal precision following axonal photostimulation.

We observed that Purkinje cell axons required longer light pulses and showed longer latencies than somata do to elicit action potentials, so we then wondered whether they may have heightened sensitivity to light perturbations. To test this, we exposed slices to ambient light and repeated our measurements. Axons required longer light pulses in this condition compared to axons maintained in low light, while there was no significant difference in the light pulse duration necessary to elicit spikes from somata held in either low light or ambient light. These results may be due to a slow recovery from inactivation induced by exposure to ambient light: ChR2(H134R) recovers from desensitization and inactivation more slowly than other engineered ChR2 variants (Lin, 2011). Since transgenically expressed ChR2 is not specifically clustered at nodes of Ranvier in myelinated axons (Figure 1; Grubb and Burrone, 2010; Arlow et al., 2013), inactivation of individual ChR2 molecules in a region with an already limited availability may greatly reduce the efficacy of a photostimulus. This axonal sensitivity supports our hypothesis that Purkinje cell axons are more affected than somata are by light perturbations in optogenetic experiments and suggests that extra care should be taken when photostimulating axons to minimize unnecessary light exposure. Finally, we confirm that the parameters which elicit well-timed single action potentials from Purkinje cell axons allow us to elicit temporally precise synaptic responses in DCN neurons with little trial-to-trial jitter. The parameters we established to best elicit single action potentials from Purkinje cell axons matched well to conditions we observed to best elicit IPSCs in target DCN neurons when focally stimulating a population of presynaptic Purkinje cells (2 or 3 ms). Given the relatively large size of our photostimulation pulse and because Purkinje cell axons bundle together in the white matter, we do not expect to have stimulated single axons, but rather, small subpopulations of Purkinje cell axons. However, further optimization of the size and location of the photostimulation pulse might allow us to reliably photostimulate individual presynaptic axons in the future. Our findings highlight the importance of empirically determining photostimulation parameters from presynaptic neurons to optimize conditions for optogenetic experiments. We expect that some of our findings, such as that axons typically require longer light pulses for similar responses to axons and are more susceptible to background ambient light, are general features that will likely be observed across cell types and recording configurations. However, the major conclusion of this work is that it is important to determine photostimulation parameters empirically when precise temporal control of action potentials is desired for optogenetic experiments.

## Data Availability Statement

The datasets generated for this study are available on request to the corresponding author.

## Ethics Statement

All animal procedures were approved by the McGill Animal Care Committee, in accordance with guidelines established by the Canadian Council on Animal Care.

## Author Contributions

KG performed the experiments and analyzed electrophysiological data. AW conceived of the project, and acquired and analyzed two-photon imaging, and electrophysiological data. Both authors designed the experiments, interpreted the data, wrote the manuscript, and have approved the final version of the manuscript and agreed to be accountable for all aspects of the work.

## Conflict of Interest

The authors declare that the research was conducted in the absence of any commercial or financial relationships that could be construed as a potential conflict of interest.

## Footnotes

1 https://imagej.nih.gov/ij/
